# Longitudinal outcomes in cryptogenic stroke patients with and without long-term cardiac monitoring for atrial fibrillation

**DOI:** 10.1016/j.hroo.2022.02.006

**Published:** 2022-02-13

**Authors:** Shadi Yaghi, Michael P. Ryan, Candace L. Gunnarsson, William Irish, Sarah C. Rosemas, Karah Neisen, Paul D. Ziegler, Matthew R. Reynolds

**Affiliations:** ∗ Department of Neurology, Brown University, Providence, Rhode Island; † Imperium Statistical Consulting, Research, Raleigh, North Carolina; ‡ Medtronic, Cardiac Rhythm & Heart Failure, Mounds View, Minnesota; § Baim Institute for Clinical Research, Economics & Quality of Life Research, Boston, Massachusetts

**Keywords:** Ambulatory electrocardiography, Atrial fibrillation, Cryptogenic stroke, Insertable cardiac monitor, Oral anticoagulation

## Abstract

**Background:**

Guidelines recommend a confirmed diagnosis of atrial fibrillation (AF) to initiate oral anticoagulation in cryptogenic stroke (CS) patients. However, the intermittent nature of AF can make detection challenging with intermittent short-term cardiac monitoring.

**Objective:**

The purpose of this retrospective cohort study was to examine post-CS utilization of cardiac monitoring and associated clinical outcomes.

**Methods:**

Adults with incident hospitalization for CS were identified in the Optum® claims database and assessed for cardiac monitoring received poststroke. Patient were stratified into those with a long-term insertable cardiac monitor (ICM) vs external cardiac monitor (ECM) only. The timing of ICM placement poststroke was treated as a time-dependent covariate. The clinical outcomes of interest were time to AF diagnosis, oral anticoagulation usage, and all-cause mortality.

**Results:**

A total of 12,994 patients met selection criteria for the analysis, of whom 1949 (15%) received an ICM and 11,045 (85%) received ECM only. In those who had received an ECM as their first monitoring modality, only 4.4% moved on to receive an ICM for longer-term monitoring. Use of ECM before ICM was associated with a longer time to AF diagnosis (median 336 vs 194 days). Compared to those with ECM only, ICM patients had a significantly lower rate of death (hazard ratio [HR] 0.70; *P* = .004), and faster time to AF diagnosis (HR 1.50; *P* <.0001) and anticoagulation initiation (HR 1.57; *P* <.0001) during follow-up of up to 5 years after CS.

**Conclusion:**

In a real-world study of CS patients, prolonged cardiac monitoring was associated with higher rates of AF detection and treatment, and higher odds of survival.

**Key Findings**

▪ In a United States commercial claims cohort of 12,944 cryptogenic stroke patients, 15% received long-term continuous monitoring with an insertable cardiac monitor (ICM) and 85% received a short-term external cardiac monitor (ECM).
▪ In those who receive an ECM as their first monitoring modality poststroke, only 4.4% moved on to receive an ICM for longer-term monitoring for atrial fibrillation (AF).
▪ Use of ECM before ICM was associated with a delay in time to AF diagnosis, from a median 194 days to 336 days.
▪ Long-term cardiac monitoring was associated with higher rates of AF detection and treatment, and lower rates of death after up to 5 years of follow-up poststroke.

## Introduction

Currently, 20%–40% of all ischemic stroke and transient ischemic attack (TIA) cases are classified as of unknown cause, or cryptogenic.^1^, ^2^, ^3^ Secondary stroke prevention in patients with cryptogenic stroke (CS) relies on identifying and treating risk factors. For instance, in patients diagnosed with atrial fibrillation (AF), oral anticoagulation is indicated to reduce the risk of recurrent stroke,^4^ whereas anticoagulation treatment is not superior to antiplatelet therapy in patients with CS in the absence of AF.^5^, ^6^, ^7^

AF is associated with a 5-fold higher risk of stroke but can easily be missed if the screening is performed only by routine short-term rhythm monitoring.^8^ In patients with CS, randomized trials have shown that prolonged cardiac monitoring leads to increased detection of AF compared to traditional monitoring methods such as the usual 24- to 48-hour in-hospital or outpatient Holter monitoring.^1^^,^^9^ This recently led to a level IIa recommendation for long-term rhythm monitoring to detect AF in patients with CS in the 2021 American Heart Association/American Stroke Association Guideline for the Prevention of Stroke in Patients with Stroke and Transient Ischemic Attack.^4^

To date, prolonged cardiac monitoring remains underutilized.^10^^,^^11^ As such, outcomes data from patients stratified by monitoring strategies are limited in the published literature. The aim of this analysis was to use real-world data to examine clinical outcomes such as time to death, AF diagnosis, use of anticoagulation, and subsequent hospitalizations in incident CS/TIA patients who receive an insertable cardiac monitoring (ICM) device vs those who only received a wearable, external cardiac monitoring (ECM) device. Additionally, to provide insight on the patients who ultimately receive ICM, we describe the clinical care pathway before ICM insertion, including the utilization of ECMs and the time delay to ICM monitoring and to AF diagnosis after a CS event.

## Methods

This was a real-world retrospective database study leveraging the Optum® (Eden Prairie, MN) de-identified Clinformatics® U.S. claims database, which contains data for privately insured and Medicare Advantage enrollees across the United States from January 1, 2007, to June 30, 2019.^12^ Because this was a noninterventional, retrospective, observational study utilizing de-identified data, informed consent was not required from the patient under an institutional review board exemption status. All aspects of this study were conducted in compliance with Health Insurance Portability and Accountability Act of 1996 (HIPAA) regulations and the HIPAA Omnibus Rule of 2013.

### Inclusion criteria

Patients were identified based on the presence of a diagnosis code for CS or TIA (International Classification of Diseases [ICD]-9 434.91, 435.9, 434.11; or ICD-10 I63.9) in the primary position during an inpatient hospitalization between January 1, 2007, and June 30, 2019. Although ICD-9 (Ninth Revision) and ICD-10 (Tenth Revision) diagnosis coding does not include explicit codes for CS, these particular codes were selected based on an analysis of the diagnosis codes used during stroke/TIA events in patients who received an ICM for the stated indication of CS (data on file). A record of ≥1 procedures indicative of an acute stroke event (see Supplemental Appendix) was required either 2 days before, during, or within 2 days after the index hospitalization, based on a published algorithm developed by Kaplan et al,^13^ evaluating rates of acute stroke and systemic embolism using the same Optum database. The population was limited to patients 18 years of age or older who had ≥12 months of continuous enrollment before their incident CS/TIA hospitalization.

Patients were excluded if any of the following occurred before their index CS/TIA hospitalization: diagnosis of AF or atrial flutter, record of an ablation for AF or atrial flutter, oral anticoagulation usage, mechanical heart valve implant, rheumatic heart disease, mitral stenosis, ischemic or dilated cardiomyopathy, ST-elevation myocardial infarction, end-stage renal disease, previous stroke/TIA, or an ICM implant. Patients also were excluded if they had any of the following procedures within 30 days before to 90 days after their index CS/TIA hospitalization, which could be indicative of a specific stroke etiology (rather than a CS event): carotid artery stenting or endarterectomy, carotid artery stenosis or occlusion, atrial septal defect, or patent foramen ovale closure. Finally, patients with any evidence of other cardiac implantable electronic devices capable of monitoring AF (pacemaker, implantable cardioverter-defibrillator, or cardiac resynchronization therapy device) at any point during baseline or follow-up were excluded as well.

### Cohort assignment

For all study participants, the analytic start time (time zero) began with the incident CS/TIA hospitalization. Patients with a record of ICM insertion on or after time zero were assigned to the ICM cohort, regardless of whether they received any external monitoring before their ICM insertion. Patients without ICM but with at least 1 ECM were assigned to the ECM cohort. The ECM cohort consisted of those with short- and long-term Holter monitors, event monitors, and mobile cardiac telemetry monitors. Patients with no ambulatory electrocardiographic monitoring (neither ICM nor ECM) were not included in this study, as they may not represent comparable cryptogenic patients.

### Data analyses: Patient characteristics

Patient demographics, index hospital stay characteristics, and patient comorbidities (measured by CHA_2_DS_2_-VASc score) were summarized for the ICM and ECM cohorts.^14^^,^^15^ The CHA_2_DS_2_-VASc score is a classification algorithm that is based on ICD-9/-10 diagnosis codes, ICD-9/-10 and CPT procedure codes, and age at index. Patients are assigned a score derived as follows: 1 point each for any record of congestive heart failure, hypertension, age 65–74 years, female gender, vascular disease, or diabetes mellitus; and 2 points for any record of stroke/TIA or age ≥75 years. For the purposes of the CHA_2_DS_2_-VASc score, vascular disease, is defined as any 1 or more of the following: coronary artery disease diagnosis, peripheral artery disease diagnosis, coronary artery bypass procedure, percutaneous coronary intervention procedure, or another coronary revascularization procedure. Characteristics of the index hospital stay were used to estimate the patient’s stroke severity, including length of stay of the index stroke hospitalization in days, intensive care unit (ICU) utilization during the index stroke hospitalization (yes vs no), and discharge status after the index hospitalization to home vs not home (ie, to a long-term care center or rehabilitation facility).

### Time to event analyses in ICM vs ECM patients

The 3 main outcomes of interest for this study were time to AF diagnosis, time to oral anticoagulation usage, and time to all-cause mortality. For time to oral anticoagulation usage, a subanalysis was performed on just those patients who received a diagnosis of AF. In order to estimate time to clinical events (time to AF diagnosis, time to anticoagulation initiation, and time to death), Cox proportional hazard models with a 5-year time horizon were used. A key explanatory variable for this research was whether a patient received an ICM on or after their incident CS/TIA diagnosis (time zero). However, the timing of an ICM implant can vary and may be delayed by varying durations after the index stroke admission (time zero). Therefore, ICM implantation was treated as a time-dependent covariate. Additional covariates incorporated in the Cox models included patient demographics (age, gender, geographic region, and payer type [commercial or Medicare Advantage]); index hospital stay characteristics indicative of stroke severity (length of stay, ICU utilization, and discharge status [home/not home]); and comorbidities as measured by the CHA_2_DS_2_-VASc score.

Each time to event model was estimated using the partial likelihood method in SAS. Model adequacy was assessed using residual diagnostics. For testing the proportional hazard assumption, the interaction with time and the independent variables was tested for significance. Additional subanalyses were performed on the time to oral anticoagulation outcome, in which a subset of patients with AF diagnosed during the follow-up period was analyzed separately.

### Characterization of monitoring pathway before ICM

To further describe the clinical pathway of CS patients who ultimately receive an ICM, before multivariable modeling we explored the pre-ICM usage of ECMs and the time from acute stroke event to ICM insertion and to AF diagnosis. Costs of external monitors were estimated utilizing 2020 U.S. national average Medicare payment rates and incorporating a 125% markup to approximate commercial payment rates.^16^

## Results

### Patient characteristics

**Figure 1:**
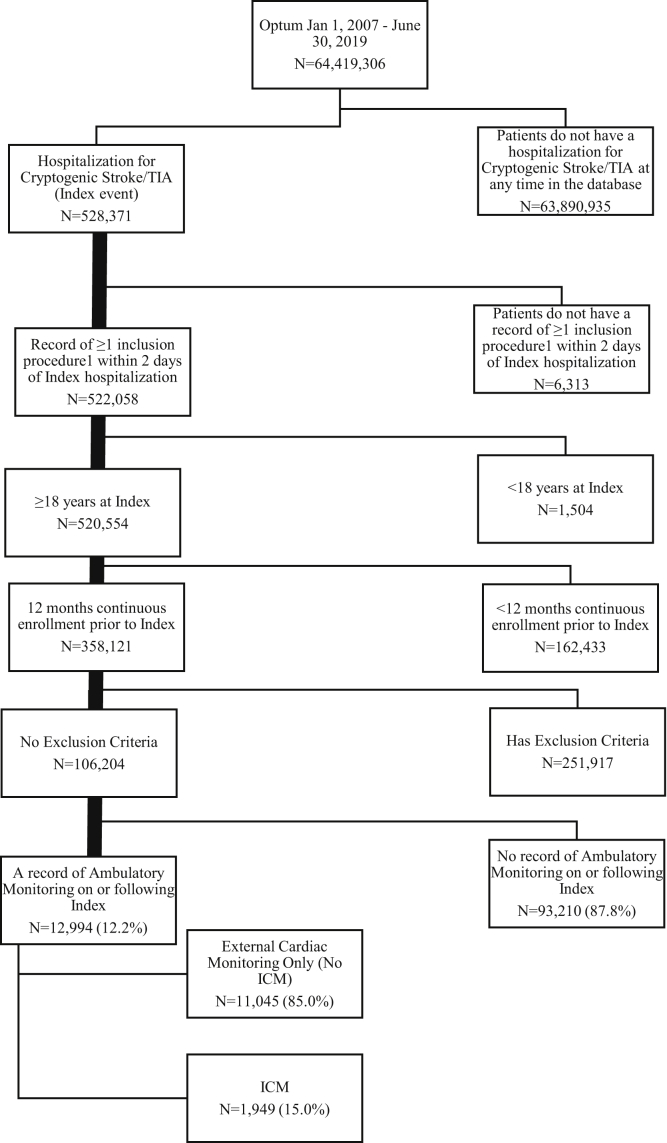
Patient selection. See Supplemental Appendix for a list of inclusion procedures. ICM = insertable cardiac monitor; TIA = transient ischemic attack.

Figure 1 shows the attrition diagram for the main analyses. Of the 12,944 patients who met inclusion/exclusion criteria, 1949 (15%) received an ICM with a median time to insertion of 26 days, and 11,045 (85%) received ECM only with a median time to ECM assignment of 69 days. Across the ECM cohort, the monitors received were of short-term Holter monitors (43%), long-term Holter monitors (9%), event monitors (32%), and mobile cardiac telemetry monitors (16%). Patients on average received 2.4 (SD 1.1) monitors, for an estimated cumulative 41 days of prescribed monitoring. Table 1 lists patient demographics, index hospital stay characteristics, and CHA_2_DS_2_-VASc score (see Table 2 for components of CHA_2_DS_2_-VASc score), which were evenly distributed between the ICM and ECM cohorts with an average age of 67 years, slightly more females than males (51% and 55%), and similar mean (± SD) CHA_2_DS_2_-VASc score (2.6 ± 1.6 and 2.6 ± 1.6), respectively. Before multivariable adjustment, mean index length of stay (8.8 vs 7.8 days; *P* = .0015) and frequency of index ICU utilization (49% vs 45%; *P* = .0006) were higher in the ICM cohort. Patients in the ECM cohort were more often discharged to home (78% vs 72%; *P* <.0001) compared to those in the ICM cohort.

**Table 1:** Patient characteristics

	ECM	ICM	*P* value
Sample size (n)	11,045	1949	
Age (y)	66.8 ± 13.5	66.9 ± 12.6	.6378
Female	6053 (54.8)	1000 (51.3)	<.0001
Follow-up duration (y)	3.17 ± 2.64	1.92 ± 1.80	<.0001
Region			.0043
Midwest	2960 (26.8)	562 (28.8)	
Northeast	1807 (16.4)	362 (18.6)	
South	4360 (39.5)	797 (40.9)	
West	1892 (17.1)	223 (11.4)	
Unknown	26 (0.2)	5 (0.3)	
Payer			.0280
Commercial	4377 (39.6)	721 (37.0)	
Medicare Advantage	6668 (60.4)	1228 (63.0)	
Index cryptogenic stroke hospitalization			
Length of stay (d)	7.8 ± 11.6	8.8 ± 12.7	.0015
ICU stay (yes)	4942 (44.7)	954 (49.0)	.0006
Discharge to home (yes)	8552 (77.7)	1388 (71.6)	<.0001
Patient comorbidity			
CHA_2_DS_2_-VASc score	2.6 ± 1.6	2.6 ± 1.6	.228

Values are given as mean ± SD or n (%) unless otherwise indicated.

ECM = external cardiac monitor; ICM = insertable cardiac monitor; ICU = intensive care unit.

**Table 2:** Components of the CHADS-VASC score

CHA_2_DS_2_-VASc component	ECM	ICM	*P* value
Age 65–74 y	3335 (30.19)	646 (33.15)	<.0001
Age 75+ y	3644 (32.99)	597 (30.63)	.0430
CHF	390 (3.53)	64 (3.28)	.6304
Hypertension	6865 (62.15)	1202 (61.67)	.7046
Diabetes	2874 (26.02)	496 (25.45)	.6149
Stroke/TIA	660 (5.98)	133 (6.82)	.1641
Vascular condition	668 (6.05)	122 (6.26)	.7573
Female	6053 (54.80)	1000 (51.31)	.0043

Values are given as n (%) unless otherwise indicated.

CHF = congestive heart failure; ECM = external cardiac monitor; ICM = insertable cardiac monitor; TIA = transient ischemic attack.

### Characterization of monitoring pathway before ICM

**Figure 2:**
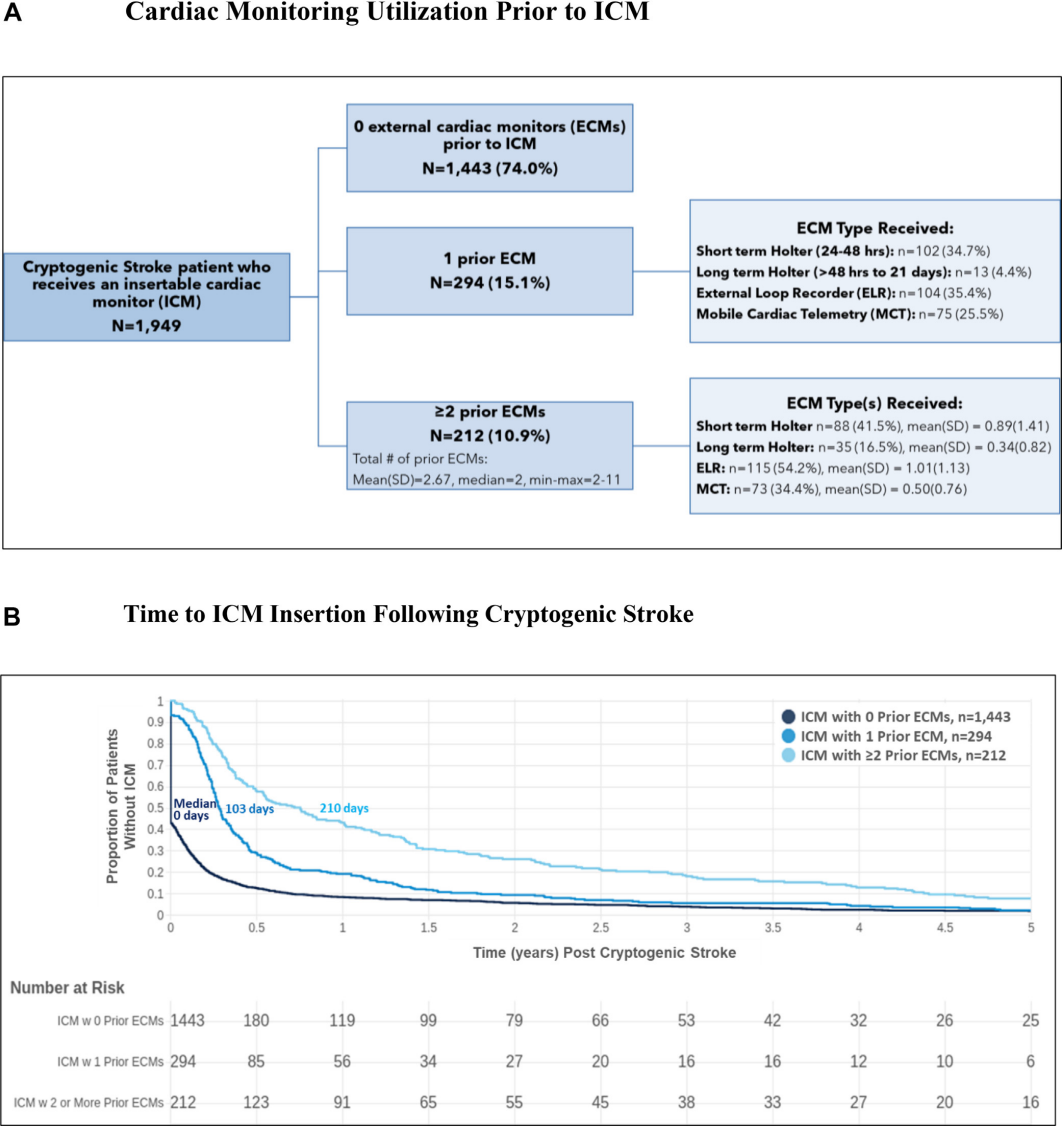
Cardiac monitoring pathway before insertable cardiac monitor (ICM) in cryptogenic stroke patients. **A:** Cardiac monitoring utilization before ICM. **B:** Time to ICM insertion after cryptogenic stroke. Median time to atrial fibrillation diagnosis was calculated for each of the ICM cohorts in B (data not shown). In ICM patients with 0 prior ECMs, median time to AF diagnosis was 194 days; in ICM patients with 1 prior ECM was 289 days; and in ICM patients with ≥2 prior ECMs was 336 days. ECM = external cardiac monitor; ELR = external loop recorder; MCT = mobile cardiac telemetry.

Descriptive summary statistics on the clinical care pathway before ICM are presented in Figure 2. Figure 2A shows a flow diagram for the clinical care pathway of patients who ultimately receive an ICM. In our sample, 1443 of these patients (76%) received the ICM as their first ambulatory cardiac monitoring modality, whereas the remaining 506 patients (26%) had a record of ECM use before ICM insertion. Of the 506 patients with prior ECMs, 294 had received 1 prior ECM distributed as follows: short-term Holter (24–48 hours) (n =102 [34.7%]); long-term Holter (>48 hours to 21 days) (n = 13 [4.4%]); external loop recorder (n = 104 [35.4%]); and mobile cardiac telemetry (n = 75 [25.5%]). These patients were slightly older (68.0 vs 66.9 years) than the overall ICM cohort but had similar CHA_2_DS_2_-VASc scores (2.5 vs 2.6). A total of 212 patients received ≥2 ECMs before ICM, with an estimated average of 51 total days of prescribed ECM monitoring before ICM implantation. The distribution of monitoring modalities used in these patients is represented in Figure 2A. Among patients with any ECM monitoring before ICM, the average cost of prior ECMs was estimated to be approximately $820 per patient.

Greater use of ECMs before ICM significantly increased the time from index stroke to ICM, from a median of 0 days in patients with no prior ECMs to 103 days and 210 days in those with 1 or ≥2 prior ECMs, respectively (Figure 2B). Likewise, median time from index stroke to AF diagnosis increased from 194 days to 289 and 336 days, respectively, when either 1 or ≥2 ECMs preceded ICM. This delay in AF diagnosis with ECM patients to ICM patients was largely driven by the delay between ECM and ICM. In patients with 1 ECM before ICM, the median amount of time between monitoring modalities was 70 days, and in patients with ≥2 ECMs before ICM the median time between first ECM and the ICM was 154 days. Overall, of the patients who received ECM as their initial cardiac monitoring modality (11,551), only 506 (4.4%) progressed to longer-term monitoring with an ICM.

### Time to event analyses in ICM vs ECM patients

**Figure 3:**
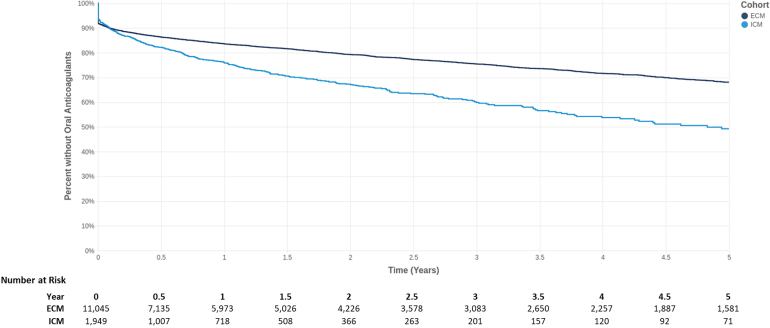
Time to anticoagulation for insertable cardiac monitor (ICM) vs external cardiac monitor (ECM).

Table 3 lists the results of all multivariable modeling for the time to event outcomes. At 5 years after their incident CS/TIA hospitalization, patients receiving ICM had a significantly lower rate of death (hazard ratio [HR] 0.70; *P* = .004), and faster time to AF diagnosis (HR 1.50; *P* <.0001) and anticoagulation usage (HR 1.57; *P* <.0001). Time from the qualifying CS to anticoagulation usage was also faster in ICM patients when the analysis was limited to patients subsequently diagnosed with AF (HR 1.12; *P* = .029). Figure 3 shows the time to anticoagulation following a patient's CS/TIA hospitalization, with an estimated 51.8% of ICM patients and 31.8% of ECM patients receiving anticoagulation at 5 years.

**Table 3:** Multivariable results: Time to clinical event

Cox regression models	Unadjusted HR	Adjusted HR	Lower CI	Upper CI	*P* value
Time to death	0.69	0.70	0.55	0.89	.0040
Time to AF diagnosis	1.51	1.50	1.40	1.60	<.0001
Time to anticoagulation	1.53	1.57	1.42	1.73	<.0001
Time to anticoagulation in patients who are diagnosed with AF	1.11	1.12	1.01	1.23	.0289

All models: ICM is a time-varying covariate. Additional covariates include patient demographics, index hospital stay characteristics indicative of stroke severity (length of stay, intensive care unit utilization, and discharge status home vs not home), and CHA_2_DS_2_-VASc score.

AF = atrial fibrillation; CI = confidence interval; HR = hazard ratio; ICM = insertable cardiac monitor.

## Discussion

Our study found that patients receiving ICM after their incident CS/TIA hospitalization had AF detected more quickly and more frequently, had an increase in anticoagulation utilization, and experienced improved survival. These findings are in line with previous studies of implantable continuous cardiac monitoring whereby “silent” AF was detected, which enabled therapeutic interventions for the mitigation of recurrent stroke risk. For example, multiple studies have demonstrated AF detection rates of around 27%–30% after 3 years of continuous monitoring following a poststroke ICM implantation and have shown that under the typical standard of care, only a fraction of those cases would have been caught, suggesting a drastically improved diagnostic yield.^1^, ^2^, ^3^^,^^17^ Direct comparisons of AF detection rates between studies, including this one, are challenging due to differences in follow-up durations, monitoring technologies, and patient demographics.^18^

An increased rate of AF detection has been associated with longer monitoring periods. The CRYSTAL AF (Cryptogenic Stroke and Underlying AF) trial showed that the rate of AF detection among patients with CS or TIA was 12.4% at 12 months for ICM patients vs 2.0% for standard of care, and at 3 years of follow-up the AF detection rate had increased to 30% in the ICM arm compared to 3.0% in controls.^1^

Tsivgoulis et al^19^ performed a meta-analysis of 4 studies of prolonged ambulatory electrocardiographic monitoring vs conventional monitoring in CS patients, totaling 1102 patients (including 2 randomized controlled trials [CRYSTAL AF and FIND-AF [Holter-Electrocardiogram-Monitoring in Patients With Acute Ischaemic Stroke], and 2 controlled observational studies of CS patients). In addition to an increased incidence of AF detection (risk ratio 2.46; 95% confidence interval [CI] 1.61–3.76) and anticoagulant initiation (risk ratio 2.07; 95% CI 1.36–3.17), this pooled analysis also showed a decreased risk of recurrent stroke (risk ratio 0.45; 95% CI 0.21–0.97) during follow-up for patients who underwent prolonged monitoring compared with patients who received conventional cardiac monitoring.^19^ A lower rate of stroke recurrence was also observed in a prospective nonrandomized clinical trial in Greece.^20^ The incidence of stroke recurrence (defined as a new neurological event recorded at least 24 hours after hospital discharge and validated by neuroimaging) in the analysis of consecutively managed patients was significantly lower in the Reveal LINQ (Medtronic, Minneapolis) group compared with the standard-of-care group (4% vs 12%; *P* = .013). A similar result was reported for the propensity score-matched analysis (ICM 2% vs standard-of-care 9%; *P* <.05).^20^

Our analysis both confirms and extends previous work in this area by showing that ICM was associated with a 54% faster time to AF diagnosis and a 57% faster time to oral anticoagulation. Additionally, for the first time, our analysis points to a possible survival benefit from the ICM strategy. This latter finding could be related to enhanced secondary prevention of stroke, although given our nonrandomized study design, the possibility that this finding is due to confounding cannot be excluded. It is possible that patients with lesser stroke or overall illness severity are more likely to be prescribed ICMs. However, we found no evidence that this was so in the measured patient characteristics, which if anything suggested that the index strokes may have been more severe in the ICM patients (slightly longer length of stay and likelihood of ICU admission). The improved survival of patients receiving ICMs also could be related to other aspects of their medical care, such as more frequent contact with providers or receiving treatment in facilities with more robust aftercare programs following an initial stroke.

Our assessment of care pathways after CS further highlights potential advantages of utilizing ICMs earlier rather than later after CS. Our data clearly demonstrate that patients managed with external monitors encounter substantial delays to AF diagnosis and the opportunity for treatment; receive ICMs much later (or not at all); and in some cases may be lost to follow-up entirely. Finally, although our observations indicate that some stroke care pathways include a trial of ECMs before ICM (likely due in part to the higher upfront cost of the ICM device), recent cost-effectiveness analysis found this approach to be overall on average cost-additive compared to use of an ICM directly after CS, due to the low likelihood of diagnosis with an initial ECM.^21^ These considerations may be important in jurisdictions with constrained health care resources, in which short- vs long-term clinical and economic implications must be weighed. Further research should focus on assessing economic and clinical implications of stroke monitoring pathways outside the United States.

### Study limitations

The most important limitation is the use of observational data, which is subject to treatment by indication bias. The limitations of this study are consistent with weaknesses in retrospective claims-based analyses, which include the reliance on billing codes and the risk of coding errors. Although we adjusted for a variety of patient characteristics and comorbidities, there may be additional underlying variables that we are not able to account for in claims data. For example, although we adjusted for the available metrics that in our clinical experience are indicative of stroke severity (length of stay, ICU utilization, and discharge status), there may be other important differences in severity that we are not able to capture. Likewise, there may be other external forces affecting these characteristics, such as the availability of health care resources, that are unrelated to the severity of the stroke. Furthermore, the retrospective design of our study and the labeling of the pathologies (CS, TIA, AF) without complete information on diagnosis details (as might be found in an electronic health record) could decrease the significance of the results and their generalizability.

Using commercial claims data, the generalizability of our findings is limited to U.S. patients with commercial insurance. Although our population is primarily of Medicare age, no fee-for-service Medicare patients are represented in our analysis (only Medicare Advantage patients insured through United Healthcare). Additionally, claims-based analyses in the area of CS are limited by the lack of explicit ICD-9 or ICD-10 diagnosis codes for stroke/TIA that remains cryptogenic after initial evaluation. However, we were able to leverage an analysis utilizing Optum linked to ICM device data to identify the 4 diagnosis codes utilized in the vast majority (93.2%) of patients receiving cardiac monitoring for a stated indication of CS (data on file). Due to the complexity and variability in cardiac monitoring pathways, our ECM cohort includes patients receiving a variety of external monitoring modalities (including short- and long-term Holter monitors, event monitors, and mobile cardiac telemetry monitors), which limits the ability to attribute outcomes to any specific monitor type. Finally, we were unable to assess rates of recurrent acute ischemic stroke events due to the lack of needed documentation in claims data, which makes it unreliable to differentiate recurrent events vs follow-up or coding of a previous event in the patient’s history. This research question is better answered utilizing detailed electronic health record data or prospective clinical studies.

## Conclusion

One of the important goals of stroke and TIA management is clarifying risk factors for potential stroke recurrence to optimize treatment. Because AF can be infrequent and intermittent in nature, detection can be challenging during the initial evaluation and with short-term monitoring in patients who experience CS. Whether it is cause or consequence of the stroke, AF is a well-established risk factor for recurrent stroke, and initiation of oral anticoagulation is indicated for stroke prophylaxis upon diagnosis of AF. In this study, we found patients with longer-term monitoring had higher rates of AF detection and treatment, and significantly higher odds of survival. Implantable cardiac devices that allow for continuous cardiac rhythm monitoring in patients with CS or TIA may aid in earlier detection of serious underlying disease such as AF, thereby enabling intervention and superior patient outcomes.
