# Supplementary figures and images for: Longitudinal outcomes in cryptogenic stroke patients with and without long-term cardiac monitoring for atrial fibrillation
Source: Heart Rhythm O2. 2022 Feb 13;3(3):223–30. doi: 10.1016/j.hroo.2022.02.006 (PMC9207734; doi:10.1016/j.hroo.2022.02.006)

**APPENDIX**

**Coding Detail**


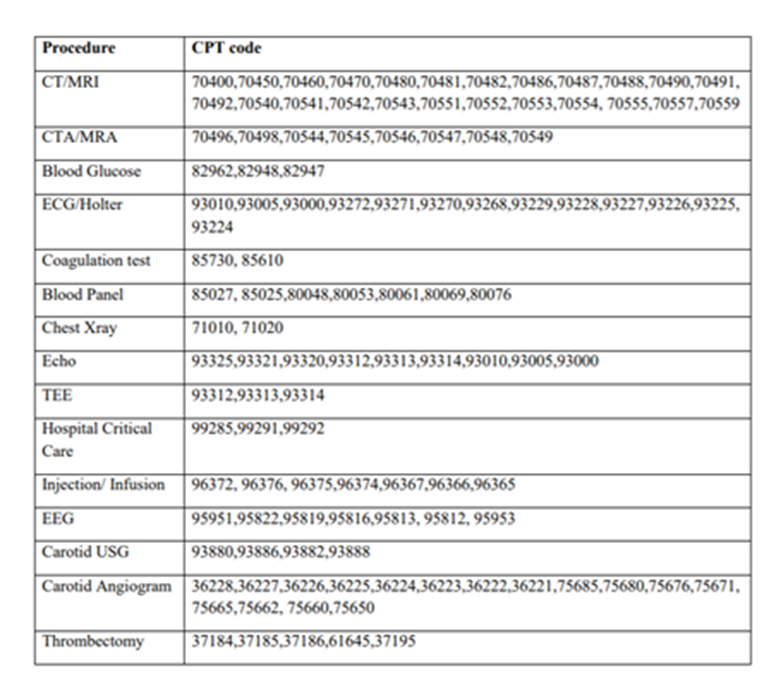

Supplement: Supplementary Appendix [file mmc1.docx]
